# Supplementary material for: Comparative genomics reveals insight into the evolutionary origin of massively scrambled genomes
Source: eLife. 2022 Nov 24;11:e82979. doi: 10.7554/eLife.82979 (PMC9797194; doi:10.7554/eLife.82979)
Supplement: Supplementary file 1. — *Sequencing data from Chen et al., 2014. **Raw reads were mapped to the MIC genome assembly by Minimap2 and Bowtie2 (Langmead and Salzberg, 2012). Average coverage was calculated with BBmap (sourceforge.net/projects/bbmap/) pileup.sh for macronuclear destined sequence-containing contigs in the MIC genome assembly. [file elife-82979-supp1.docx]

**Supplementary File 1.** Sequencing depth statistics for MIC genome assemblies

|  | ***Oxytricha trifallax**** | ***Tetmemena sp.*** | ***Euplotes woodruffi*** |
| --- | --- | --- | --- |
| Illumina coverage (X) | 118 | 69 | 190 |
| PacBio coverage (X) | 34 | 28 | 29 |
| Nanopore coverage (X) | - | - | 62 |

*Sequencing data from Chen et al. (1).

**Raw reads were mapped to the MIC genome assembly by Minimap2 and Bowtie2 (97). Average coverage was calculated with BBmap ([sourceforge.net/projects/bbmap/](http://sourceforge.net/projects/bbmap/)) pileup.sh for MDS-containing contigs in the MIC genome assembly.
