# Supplementary material for: Comparative genomics reveals insight into the evolutionary origin of massively scrambled genomes
Source: eLife. 2022 Nov 24;11:e82979. doi: 10.7554/eLife.82979 (PMC9797194; doi:10.7554/eLife.82979)
Supplement: Supplementary file 2. — Repeat content of the three genomes, as annotated by Repeatmasker (Smit et al., 2013) with additional manual annotation of Telomere-Bearing Element (TBE)/Transposon of Euplotes crassus (TEC) elements. The numbers may differ from Figure 2A–C because some repeats are assigned as other germline micronucleus (MIC) categories in the pie charts (Methods). For example, a MIC region which is both an internally eliminated sequence (IES) and satellite, is assigned as IES in Figure 2A–C, but is counted as a satellite in this table. [file elife-82979-supp2.docx]

**Supplementary File 2.** Subcategories of repeat content in the three species.

|  |  | ***Oxytricha trifallax*** | ***Tetmemena sp.*** | ***Euplotes woodruffi*** |
| --- | --- | --- | --- | --- |
| Transposable elements | Class 2 TBE/Tec | 15.4% | 1.9% | 2.3% |
|  | Class 2 cut-and-paste DNA transposons (excluding TBE/Tec) | 2.8% | 0.9% | 2.8% |
|  | Class 1 LTR | 1.2% | 1.1% | 0.7% |
|  | Class 1 LINE | 1.1% | 0.1% | 0.2% |
|  | Class 2 *Helitron* | 0.5% | 0.0% | 0.1% |
|  | Class 1 SINE | 0.1% | 0.0% | 0.0% |
|  | Unclassified | 27.4% | 14.9% | 15.9% |
| Tandem repeats | Satellites | 0.1% | 0.1% | 0.0% |
|  | Simple repeats | 1.4% | 1.0% | 0.5% |
|  | Low-complexity | 0.4% | 0.4% | 0.2% |
| Total percentage of the MIC genome assembly | | 49.6% | 20.2% | 22.5% |
