# Supplementary material for: Comparative genomics reveals insight into the evolutionary origin of massively scrambled genomes
Source: eLife. 2022 Nov 24;11:e82979. doi: 10.7554/eLife.82979 (PMC9797194; doi:10.7554/eLife.82979)
Supplement: Supplementary file 3. — * Differs from 10,109 in Chen et al. (Chen and Landweber, 2016) because we used different versions of BLAST and custom python scripts to identify complete TBEs (see Methods). [file elife-82979-supp3.docx]

**Supplementary File 3.** TBE/Tec ORFs in three species

|  | ***Oxytricha trifallax*** | ***Tetmemena sp.*** | ***Euplotes woodruffi*** |
| --- | --- | --- | --- |
| ORF1 | 21,433 | 1645 | 1202 |
| ORF2 | 16,816 | 723 | 112 |
| ORF3 | 19,582 | 641 | 245 |
| complete | 9313* | 48 | 74 |

* Differs from 10,109 in Chen et al. (44) because we used different versions of BLAST and custom python scripts to identify complete TBEs (See Methods).
