# Supplementary material for: Comparative genomics reveals insight into the evolutionary origin of massively scrambled genomes
Source: eLife. 2022 Nov 24;11:e82979. doi: 10.7554/eLife.82979 (PMC9797194; doi:10.7554/eLife.82979)
Supplement: Supplementary file 4. — * Ciliate database is generated by extracting all protein sequences in phylum Ciliophora (taxid: 5878) from NR database. [file elife-82979-supp4.docx]

Supplementary File 4. Orthology among scrambled and nonscrambled genes in the three species

| ciliate database* | no | 17.88% | 26.43% | 31.33% | 21.09% | 32.56% | 31.20% |
| --- | --- | --- | --- | --- | --- | --- | --- |
|  | yes | 82.12% | 73.57% | 68.67% | 78.91% | 67.44% | 68.80% |
| *Euplotes woodruffi* | No ortholog | 72.68% | 74.96% |  | 76.04% | 79.46% |  |
|  | nons-crambled | 23.89% | 21.89% |  | 21.74% | 18.74% |  |
|  | scrambled | 3.43% | 3.14% |  | 2.22% | 1.81% |  |
| *Tetmemena sp.* | No ortholog | 20.12% |  | 83.29% | 21.69% |  | 82.58% |
|  | non-scrambled | 28.62% |  | 12.27% | 72.43% |  | 14.25% |
|  | scrambled | 51.26% |  | 4.45% | 5.88% |  | 3.17% |
| *Oxytricha trifallax* | No ortholog |  | 27.97% | 83.12% |  | 33.26% | 82.98% |
|  | non-scrambled |  | 21.77% | 11.98% |  | 60.39% | 13.58% |
|  | scrambled |  | 50.25% | 4.90% |  | 6.36% | 3.44% |
|  |  | *Oxytricha* scrambled genes (3613) | *Tetmemena* scrambled genes (3371) | *E. woodruffi* scrambled genes (2429) | *Oxytricha* nonscrambled genes (19454) | *Tetmemena* nonscrambled genes (21377) | *E. woodruffi* nonscrambled genes (30950) |

* Ciliate database is generated by extracting all protein sequences in phylum Ciliophora (taxid: 5878) from NR database.
