# Supplementary material for: Comparative genomics reveals insight into the evolutionary origin of massively scrambled genomes
Source: eLife. 2022 Nov 24;11:e82979. doi: 10.7554/eLife.82979 (PMC9797194; doi:10.7554/eLife.82979)
Supplement: Supplementary file 5. — The (i,j) cell shows the number of genes in species i with an ortholog in species j. * Genes with no ortholog detected by OrthoFinder (Emms and Kelly, 2019) in the other two species. [file elife-82979-supp5.docx]

**Supplementary File 5.** Summary of orthologs in each pair of species

|  | ***Oxytricha*** | ***Tetmemena*** | ***E. woodruffi*** | **Species-specific genes*** | **ciliate database** | **total number of genes (telomeric contigs)** |
| --- | --- | --- | --- | --- | --- | --- |
| ***Oxytricha*** |  | 17847 | 5159 | 4839 | 18258 | 23067 |
| ***Tetmemena*** | 16347 |  | 4811 | 7885 | 16831 | 24748 |
| ***E. woodruffi*** | 5285 | 5494 |  | 27472 | 22825 | 33379 |
