# Supplementary material for: Comparative genomics reveals insight into the evolutionary origin of massively scrambled genomes
Source: eLife. 2022 Nov 24;11:e82979. doi: 10.7554/eLife.82979 (PMC9797194; doi:10.7554/eLife.82979)
Supplement: Supplementary file 6. [file elife-82979-supp6.docx]

**Supplementary File 6.** More scrambled MAC contigs contain at least one paralogous MDS that may be involved in alternative rearrangement.

|  | ***O. trifallax*** | ***Tetmemena sp.*** | ***E. woodruffi*** |
| --- | --- | --- | --- |
| Paralogous MDSs on scrambled contigs | 694 | 894 | 441 |
| Paralogous MDSs on nonscrambled contigs | 1340 | 949 | 524 |
| Scrambled contigs with paralogous MDSs (vs. total scrambled contigs) | 304 (2852) | 270 (2556) | 223 (1913) |
| Nonscrambled contigs with paralogous MDSs (vs. total nonscrambled contigs) | 809 (17484) | 435 (18609) | 422 (26381) |
| *p-*value (chi-square test) for contig numbers | 4e-39 | 1e-104 | 4e-177 |
