# Supplementary material for: Comparative genomics reveals insight into the evolutionary origin of massively scrambled genomes
Source: eLife. 2022 Nov 24;11:e82979. doi: 10.7554/eLife.82979 (PMC9797194; doi:10.7554/eLife.82979)
Supplement: Supplementary file 7. [file elife-82979-supp7.docx]

**Supplementary File 7.** MDS-IES pairs share homologous sequences in the three species (related to Figure 4 - figure supplement 2).

​​

|  | *E. woodruffi* | | *Oxytricha* | | | *Tetmemena* | | |
| --- | --- | --- | --- | --- | --- | --- | --- | --- |
|  |  | | recent -> ancestral | | | recent -> ancestral | | |
|  | similar length^a^ | all | Group 1^b^ | Group 2 | Group 3 | Group 1 | Group 2 | Group 3 |
| # MDS-IES pairs | 248 | 504 | 370 | 4055 | 520 | 1005 | 3222 | 369 |
| # pairs with a homologous core sequence | 224 | 272 | 17 | 20 | 2 | 16 | 20 | 2 |
| Ratio | 90.32% | 53.97% | 4.59% | 0.49% | 0.38% | 1.59% | 0.62% | 0.54% |
| G test with Williams correction  *p-value* | - | - | 9.09e-09 | | | 0.022 | | |

^a^ MDS length is between 0.8 * IES length and 1.2 * IES length.

^b^ MDS-IES pairs were dated on a phylogenetic tree as shown in Figure 4 - figure supplement 2C.
