# Supplementary material for: Comparative genomics reveals insight into the evolutionary origin of massively scrambled genomes
Source: eLife. 2022 Nov 24;11:e82979. doi: 10.7554/eLife.82979 (PMC9797194; doi:10.7554/eLife.82979)
Supplement: Supplementary file 8. [file elife-82979-supp8.docx]

**Supplementary File 8.** Genes with expression support in the three species

|  | Scrambled genes with any expression | Nonscrambled genes with any expression |
| --- | --- | --- |
| *Oxytricha trifallax* | 89.14% | 89.76% |
| *Tetmemena sp.* | 97.98% | 97.08% |
| *Euplotes woodruffi* | 82.46% | 93.65% |
