# Supplementary material for: Comparative genomics reveals insight into the evolutionary origin of massively scrambled genomes
Source: eLife. 2022 Nov 24;11:e82979. doi: 10.7554/eLife.82979 (PMC9797194; doi:10.7554/eLife.82979)
Supplement: Supplementary file 9. [file elife-82979-supp9.docx]

**Supplementary File 9.** Presence of conserved pointers in three species, with Monte Carlo simulations

|  |  | *Oxytricha* vs. *Tetmemena* | *Oxytricha* vs. *Euplotes* | *Tetmemena* vs. *Euplotes* |
| --- | --- | --- | --- | --- |
| Observed # of conserved pointers | Number of orthologs | 1345 | 51 | 52 |
|  | Number of pointer pairs | 4448 | 56 | 58 |
|  | Pointers conserved in 3 species | 23 | | |
| Expectations (Monte Carlo simulations) | Number of orthologs | 697 | 52 | 57 |
|  | *p*-value | <0.001 | 0.59 | 0.738 |
|  | Number of pointer pairs | 1781 | 57 | 62 |
|  | *p*-value | <0.001 | 0.6 | 0.783 |
|  | Pointers conserved in 3 species | 6.7 | | |
|  | *p*-value | <0.001 | | |
