# Supplementary material for: Comparative genomics reveals insight into the evolutionary origin of massively scrambled genomes
Source: eLife. 2022 Nov 24;11:e82979. doi: 10.7554/eLife.82979 (PMC9797194; doi:10.7554/eLife.82979)
Supplement: Supplementary file 10. [file elife-82979-supp10.docx]

**Supplementary File 10.** Scrambled pointers are more conserved than nonscrambled pointers.

|  | *Oxytricha* | | *Tetmemena* | |
| --- | --- | --- | --- | --- |
|  | scrambled | nonscrambled | scrambled | nonscrambled |
| Conserved | 1442 | 2715 | 1412 | 2549 |
| Unconserved | 611 | 6091 | 801 | 8046 |
| chi-square test  *p*-value | 1e-239 | | 5e-296 | |
