# Supplementary material for: Comparative genomics reveals insight into the evolutionary origin of massively scrambled genomes
Source: eLife. 2022 Nov 24;11:e82979. doi: 10.7554/eLife.82979 (PMC9797194; doi:10.7554/eLife.82979)
Supplement: Supplementary file 11. [file elife-82979-supp11.docx]

**Supplementary File 11.** Most pointers conserved in position are different in sequence

|  | *Oxytricha*-*Tetmemena* pointers |
| --- | --- |
| Same | 389 |
| pointer 1 contained in pointer 2 | 320 |
| pointer 2 contained in pointer 1 | 250 |
| Different | 3489 |
| Total | 4448 |
