# Supplementary material for: Comparative genomics reveals insight into the evolutionary origin of massively scrambled genomes
Source: eLife. 2022 Nov 24;11:e82979. doi: 10.7554/eLife.82979 (PMC9797194; doi:10.7554/eLife.82979)
Supplement: Supplementary file 12. [file elife-82979-supp12.docx]

**Supplementary File 12.** Intron-IES conversion comparison in three species and Monte Carlo simulations

|  | *Euplotes* intron – *Oxytricha*/*Tetmemena* IES | | | *Tetmemena* intron – *Oxytricha*/*Euplotes* IES | | | *Oxytricha* intron – *Tetmemena*/*Euplotes* IES | | |
| --- | --- | --- | --- | --- | --- | --- | --- | --- | --- |
|  | expected | observed | *p*-value | expected | observed | *p*-value | expected | observed | *p*-value |
| Positions | 34 | 103 | <0.001 | 1.4 | 1 | 0.767 | 1.8 | 0 | - |
|  | *Euplotes* IES – *Oxytricha*/*Tetmemena* intron | | | *Tetmemena* IES – *Oxytricha*/*Euplotes* intron | | | *Oxytricha* IES – *Tetmemena*/*Euplotes* intron | | |
|  | expected | observed | *p*-value | expected | observed | *p*-value | expected | observed | *p*-value |
| Positions | 2.4 | 24 | <0.001 | 11 | 34 | <0.001 | 7.6 | 12 | 0.098 |
