# Supplementary material for: Comparative genomics reveals insight into the evolutionary origin of massively scrambled genomes
Source: eLife. 2022 Nov 24;11:e82979. doi: 10.7554/eLife.82979 (PMC9797194; doi:10.7554/eLife.82979)
Supplement: Supplementary file 13. [file elife-82979-supp13.docx]

**Supplementary File 13.** Pairwise intron-IES conversion comparisons and Monte Carlo simulations

|  | ***Oxytricha* intron–*Tetmemena* IES** | | | ***Oxytricha* intron –**  ***Euplotes* IES** | | | ***Tetmemena* intron–**  ***Euplotes* IES** | | |
| --- | --- | --- | --- | --- | --- | --- | --- | --- | --- |
|  | expected | observed | *p*-value | expected | observed | *p*-value | expected | observed | *p*-value |
| Positions | 416 | 463 | 0.008 | 29 | 31 | 0.402 | 26 | 26 | 0.55 |
|  | ***Tetmemena* intron– *Oxytricha* IES** | | | ***Euplotes* intron –**  ***Oxytricha* IES** | | | ***Euplotes* intron – *Tetmemena* IES** | | |
|  | expected | observed | *p*-value | expected | observed | *p*-value | expected | observed | *p*-value |
| Positions | 308 | 247 | 1 | 288 | 277 | 0.745 | 349 | 347 | 0.559 |
