# Supplementary material for: Comparative genomics reveals insight into the evolutionary origin of massively scrambled genomes
Source: eLife. 2022 Nov 24;11:e82979. doi: 10.7554/eLife.82979 (PMC9797194; doi:10.7554/eLife.82979)
Supplement: Supplementary file 14. [file elife-82979-supp14.docx]

**Supplementary File 14.** PCR primers for validation of the Russian doll region in *Tetmemena* MIC DNA (Figure 6A)

| Primer | Sequence |
| --- | --- |
| 1 F | CATTCTTATTTCCCTTCATTTGTTTC |
| 1 R | CTTTCAATCTATTAAGGAGTATCTC |
| 2 F | GGCTAAAGTAAGAATATTTTATTTGAAG |
| 2 R | CAATAAATGCATGAGTTTAAATAATATCG |
| 3 F | GAGCAGGCTTGATTCAACAAAATC |
| 3 R | CATTTAAATCTTAAAAAGAGATTTTTCC |
| 4 F | CACCTACTAACTTTGAAAGACAAAG |
| 4 R | CATAGAGCTGATTTAATAACTTCATATC |
| 5 F | CTGCCCAGTCCAAATTTAAATCAAT |
| 5 R | GTTGTTAATATTTCCTTACTTATTAC |
| 6 F | TATAGCAGCTAAGGAAATCAAAATTAG |
| 6 R | CTTTTAAAGAAGGGGACAAATAACAAG |
| 7 F | CTTACCAAAAGCATTATTTAAGATGC |
| 7 R | GGATCTAATAGTGTAATAAATATCTTG |
| 8 F | ACTTACACTCAATTTAAAACAGATTG |
| 8 R | CAGATTTTCCTCCATGTTTAAAAGTC |
| 9 F | GTTCACTATGAATCTAGAAGAGATTTAAG |
| 9 R | CTCTTTCCTGATTATTCAAGGAAAAATAG |
| 10 F | CATAAATCAGACTAAAAAATTCATGC |
| 10 R | CAAAATAGATATGATAATGTCAGAAATG |
| 11 F | CTTATGTCTCTAGTAAAAATAATTATAAAC |
| 11 R | GGCATTTCATAGATCTTACTTTAAC |
| 40 F | CTCGGTATACATATATAACTATG |
