# Supplementary material for: Comparative genomics reveals insight into the evolutionary origin of massively scrambled genomes
Source: eLife. 2022 Nov 24;11:e82979. doi: 10.7554/eLife.82979 (PMC9797194; doi:10.7554/eLife.82979)
Supplement: MDAR checklist [file elife-82979-mdarchecklist1.docx]

**Materials Design Analysis Reporting (MDAR)**

**Checklist for Authors**

The [MDAR framework](https://osf.io/xfpn4/) establishes a minimum set of requirements in transparent reporting mainly applicable to studies in the life sciences.

*eLife* asks authors to **provide detailed information within their article** to facilitate the interpretation and replication of their work. Authors can also upload supporting materials to comply with relevant reporting guidelines for health-related research (see [EQUATOR Network](http://www.equator-network.org/%20)), life science research (see the [BioSharing Information Resource](http://biosharing.org/)), or animal research (see the [ARRIVE Guidelines](http://www.plosbiology.org/article/info:doi/10.1371/journal.pbio.1000412) and the [STRANGE Framework](https://doi.org/10.1038/d41586-020-01751-5); for details, see *eLife*’s [Journal Policies](https://reviewer.elifesciences.org/author-guide/journal-policies)). Where applicable, authors should refer to any relevant reporting standards materials in this form.

For all that apply, please note **where in the article** the information is provided. Please note that we also collect information about data availability and ethics in the submission form.

**Materials:**

| **Newly created materials** | **Indicate where provided: section/figure legend** | **N/A** |
| --- | --- | --- |
| The manuscript includes a dedicated "materials availability statement" providing transparent disclosure about availability of newly created materials including details on how materials can be accessed and describing any restrictions on access. |  | N/A |
|  |  |  |
| **Antibodies** | **Indicate where provided: section/figure legend** | **N/A** |
| For commercial reagents, provide supplier name, catalogue number and [RRID](https://scicrunch.org/resources), if available. |  | N/A |
|  |  |  |
| **DNA and RNA sequences** | **Indicate where provided: section/figure legend** | **N/A** |
| DNA primers for validation of Russian Doll locus in *Tetmemena sp.* | Primer sequences are provided in Supplementary File 14. Details are described in Methods section “PCR validation of Russian doll locus” |  |
| DNA sequences of *Tetmemena sp.* micronucleus, including paired-end Illumina reads and PacBio reads. | Methods section “DNA collection and sequencing of *Tetmemena sp.*” |  |
| DNA sequences of *Euplotes woodruffi* micronucleus, including paired-end Illumina reads, PacBio and Nanopore reads. | Methods section “DNA collection and sequencing for *E. woodruffi*“ |  |
| Paired-end Illumina DNA sequences of *Euplotes woodruffi* macronucleus. | Methods section “DNA collection and sequencing for *E. woodruffi*” |  |
| RNA-seq of asexually growing *Euplotes woodruffi* and *Tetmemena sp* | Methods section “RNA sequencing of *E. woodruffi and Tetmemena sp.*” |  |
|  |  |  |
| **Cell materials** | **Indicate where provided: section/figure legend** | **N/A** |
| *Tetmemena sp.* strain SeJ-2015 | Methods section “DNA collection and sequencing of *Tetmemena sp.*” |  |
| *Euplotes woodruffi* strain Iz01 | Methods section “DNA collection and sequencing for *E. woodruffi*” |  |
|  |  |  |
| **Experimental animals** | **Indicate where provided: section/figure legend** | **N/A** |
| Laboratory animals or Model organisms: Provide species, strain, sex, age, genetic modification status. Provide accession number in repository OR supplier name, catalog number, clone number, OR RRID. |  | N/A |
| Animal observed in or captured from the field: Provide species, sex, and age where possible. |  | N/A |
|  |  |  |
| **Plants and microbes** | **Indicate where provided: section/figure legend** | **N/A** |
| Plants: provide species and strain, ecotype and cultivar where relevant, unique accession number if available, and source (including location for collected wild specimens). |  | N/A |
| Microbes:  *Tetmemena sp.* strain SeJ-2015  *Euplotes woodruffi* strain Iz01 | Methods sections “DNA collection and sequencing of *Tetmemena sp.*” and “DNA collection and sequencing for *E. woodruffi*” |  |
|  |  |  |
| **Human research participants** | **Indicate where provided: section/figure legend) or state if these demographics were not collected** | **N/A** |
| If collected and within the bounds of privacy constraints report on age, sex, gender and ethnicity for all study participants. |  | N/A |

**Design:**

| **Study protocol** | **Indicate where provided: section/figure legend** | **N/A** |
| --- | --- | --- |
| If the study protocol has been pre-registered, provide DOI. For clinical trials, provide the trial registration number OR cite DOI. |  | N/A |
|  |  |  |
| **Laboratory protocol** | **Indicate where provided: section/figure legend** | **N/A** |
| Micronuclei of *Tetmemena sp.* are isolated following the protocol in <https://doi.org/10.1016/0092-8674(76)90256-7> | Methods section “DNA collection and sequencing of *Tetmemena sp.*” |  |
|  |  |  |
| **Experimental study design (statistics details) *** | | |
| **For in vivo studies: State whether and how the following have been done** | **Indicate where provided: section/figure legend. If it could have been done, but was not, write “not done”** | **N/A** |
| Sample size determination |  | N/A |
| Randomisation |  | N/A |
| Blinding |  | N/A |
| Inclusion/exclusion criteria |  | N/A |
|  |  |  |
| **Sample definition and in-laboratory replication** | **Indicate where provided: section/figure legend** | **N/A** |
| Three biological replicates of poly(A)-enriched RNA were isolated from asexually growing *E. woodruffi* and *Tetmemena sp.* cells | Methods section “RNA sequencing of *E. woodruffi* and *Tetmemena sp.*” |  |
|  |  |  |
| **Ethics** | **Indicate where provided: section/submission form** | **N/A** |
| Studies involving human participants: State details of authority granting ethics approval (IRB or equivalent committee(s), provide reference number for approval. |  | N/A |
| Studies involving experimental animals: State details of authority granting ethics approval (IRB or equivalent committee(s), provide reference number for approval. |  | N/A |
| Studies involving specimen and field samples: State if relevant permits obtained, provide details of authority approving study; if none were required, explain why. |  | N/A |
|  |  |  |
| **Dual Use Research of Concern (DURC)** | **Indicate where provided: section/submission form** | **N/A** |
| If study is subject to dual use research of concern regulations, state the authority granting approval and reference number for the regulatory approval. |  | N/A |

**Analysis:**

| **Attrition** | **Indicate where provided: section/figure legend** | **N/A** | |
| --- | --- | --- | --- |
| Genes with a coefficient of variation of TPM (transcripts per million) higher than 1 were excluded from the expression level comparison. The excluded genes have a high noise-to-signal ratio, and thus the mean may not represent real expression levels. | Figure 4 - figure supplement 3 |  | |
|  |  |  | |
| **Statistics** | **Indicate where provided: section/figure legend** | **N/A** | |
| To test the hypothesis if similar numbers of scrambled and nonscrambled chromosomes possess a paralogous MDS, we used a chi-square test. The p-value is <1e-10 so the null hypothesis is rejected. | Results section “Scrambled genes are associated with local paralogy” and Supplementary File 6 |  | |
| To test the hypothesis if the pointer location is similarly conserved for scrambled vs. nonscrambled pointers, we used a chi-square test. The p-value is <1e-10 so the null hypothesis is rejected. | Results section “*Oxytricha* and *Tetmemena* share conserved DNA rearrangement junctions” and Supplementary File 10 |  | |
| To test if pointer (or intron) locations are genuinely conserved versus coincidental matching by chance, we performed Monte Carlo tests, as also used to study intron conservation (ref. 58). | Methods section “Ortholog comparison pipeline and Monte Carlo simulations”, Supplementary File 9, Supplementary File 12 and Supplementary File 13. |  | |
| To compare the size of orthogroups between those containing scrambled genes vs. those only containing nonscrambled genes, we used a Mann-Whitney U test. The p-value is <1e-5 so the null hypothesis that orthogroup size is similar is rejected. | Results section “Scrambled genes are associated with local paralogy” and Figure 4 |  | |
| We compared the expression level of scrambled genes vs. nonscrambled genes via a Mann-Whitney U test. | Results section “Scrambled genes are associated with local paralogy” and Figure 4 - figure supplement 3 |  | |
| We tested if the length of MDS is correlated with the length of corresponding IES (which shares the same pointers) from odd-even loci by Spearman correlation test. | Results section “Scrambled genes are associated with local paralogy”, rho and p-values in Figure 4 - figure supplement 2 |  | |
| To test if similar numbers of MDS-IES pairs from odd-even loci, which emerged at different stages during evolution, present sequence homology, we used a G test with Williams correction. | Supplementary File 7 |  | |
|  |  |  |  |
| **Data availability** | **Indicate where provided: section/submission form** | **N/A** |  |
| New Datasets:  DNA-seq reads and genome assemblies have been submitted to NCBI under Bioprojects PRJNA694964 (*Tetmemena sp.*) and PRJNA781979 (*Euplotes woodruffi*). Three replicates of RNA-seq reads for vegetative cells have been submitted to NCBI under accession numbers of SRR21815378, SRR21815379, SRR21815378 for *E. woodruffi* and SRR21817702, SRR21817703 and SRR21817704 for *Tetmemena sp.*.  Genbank accession numbers for genomes are JAJKFJ000000000 (*Tetmemena sp.* Micronucleus genome), JAJLLS000000000 (*Euplotes woodruffi* Micronucleus genome), and JAJLLT000000000 (*Euplotes woodruffi* Macronucleus genome).  MDSs annotations for three species are available at <http://knot.math.usf.edu/mds_ies_db/2022/>  Genbank accession IDs are currently under embargo. Genome assemblies and reads will be released automatically when the paper is published.  Reviewer's links:  *Tetmemena sp.*: <https://dataview.ncbi.nlm.nih.gov/object/PRJNA694964?reviewer=cosbd25h2t4pasmr64sfu2f637>  *Euplotes woodruffi:*  MIC genome: <https://dataview.ncbi.nlm.nih.gov/object/PRJNA694926?reviewer=mp4qvrtbno4okok2al4i9pp3b3>  MAC genome  <https://dataview.ncbi.nlm.nih.gov/object/PRJNA781602?reviewer=qs15psf7efr5hiqoldoomaprq4> | Data availability section |  |  |
| Previous datasets:  *Oxytricha trifallax* expression data are from NCBI GSM2475111, GSM2475112 and GSM2475113. | Data availability section |  |  |
|  |  |  |  |
| **Code availability** | **Indicate where provided: section/figure legend** | **N/A** |  |
| Custom scripts are public on <https://github.com/yifeng-evo/Oxytricha_Tetmemena_Euplotes> | Data availability section |  |  |

**Reporting:**

The MDAR framework recommends adoption of discipline-specific guidelines, established and endorsed through community initiatives.

| **Adherence to community standards** | **Indicate where provided: section/figure legend** | **N/A** |
| --- | --- | --- |
| State if relevant guidelines (e.g., ICMJE, MIBBI, ARRIVE, STRANGE) have been followed, and whether a checklist (e.g., CONSORT, PRISMA, ARRIVE) is provided with the manuscript. |  | N/A |

* We provide the following guidance regarding transparent reporting and statistics; we also refer authors to [Ten common statistical mistakes to watch out for when writing or reviewing a manuscript](https://doi.org/10.7554/eLife.48175).

**Sample-size estimation**

- You should state whether an appropriate sample size was computed when the study was being designed
- You should state the statistical method of sample size computation and any required assumptions
- If no explicit power analysis was used, you should describe how you decided what sample (replicate) size (number) to use

**Replicates**

- You should report how often each experiment was performed
- You should include a definition of biological versus technical replication
- The data obtained should be provided and sufficient information should be provided to indicate the number of independent biological and/or technical replicates
- If you encountered any outliers, you should describe how these were handled
- Criteria for exclusion/inclusion of data should be clearly stated
- High-throughput sequence data should be uploaded before submission, with a private link for reviewers provided (these are available from both GEO and ArrayExpress)

**Statistical reporting**

- Statistical analysis methods should be described and justified
- Raw data should be presented in figures whenever informative to do so (typically when N per group is less than 10)
- For each experiment, you should identify the statistical tests used, exact values of N, definitions of center, methods of multiple test correction, and dispersion and precision measures (e.g., mean, median, SD, SEM, confidence intervals; and, for the major substantive results, a measure of effect size (e.g., Pearson's r, Cohen's d)
- Report exact p-values wherever possible alongside the summary statistics and 95% confidence intervals. These should be reported for all key questions and not only when the p-value is less than 0.05.

**Group allocation**

- Indicate how samples were allocated into experimental groups (in the case of clinical studies, please specify allocation to treatment method); if randomization was used, please also state if restricted randomization was applied
- Indicate if masking was used during group allocation, data collection and/or data analysis
